# The effect of whole body vibration on the sprint ability of Korean national bobsled and skeleton athletes

**DOI:** 10.1371/journal.pone.0258353

**Published:** 2021-10-08

**Authors:** Seok-Ki Min, Kwangkyu Lee, Seung-Taek Lim

**Affiliations:** 1 Department of Sport Science, Korea Institute of Sport Science (KISS), Seoul, Republic of Korea; 2 Department of Exercise Rehabilitation, Jeonju Kijeon College, Jeonju, Republic of Korea; 3 Olympic Studies Center, Kangwon National University, Gangwon-do, Republic of Korea; Universita degli Studi di Milano, ITALY

## Abstract

This study aims to find out whether whole body vibration has an effect on the sprint ability to shorten the time of bobsled-skeleton athletes. Seventeen bobsled-skeleton athletes (male = 11, female = 6) were recruited from Korea Winter Olympics National Team. Participants were randomly assigned to either a sprint immediately after whole body vibration or a sprint without whole body vibration protocol during two separate visits by a period of 3 months. To evaluate the effects of the sprint ability, measurements were performed 60-m sprint recorded test. In males, at the 45m (p<0.05) significant faster sprint section record after WBV more than Non-WBV. In females, at the 15m (p<0.05), 30m (p<0.01), 45m (p<0.05), and 60m (p<0.05) significant faster sprint section record after WBV more than Non-WBV. In males, at the 30m (p<0.05), 45m (p<0.05), and 50m (p<0.05) significant faster sprint cumulative record after WBV more than Non-WBV. In females, at the 15m (p<0.05), 30m (p<0.05), 45m (p<0.01), 50m (p<0.01), and 60m (p<0.01) significant faster sprint cumulative record after WBV more than Non-WBV. This study indicated that significant faster after WBV more than Non-WBV in males and females bobsled-skeleton athletes.

## 1. Introduction

Like the bobsled-skeleton, the sledding event starts and ends within 6 seconds; however, these steps make the greatest contribution to race performance [1]. At the start stage, the athletes must push the sled and push the sled about 55 m within 60 seconds of the start signal to start the race while running at full speed [2]. According to the International Bobsleigh & Skeleton Federation (IBSF) 2016–17 season 8th World Cup match results report, it can be seen that the top 80% of the athletes in the start record are in the top 10 of the final rankings [3]. As such, the ability to start and ride a sled is very important for bobsled and skeleton athletes because the start of bobsled-skeleton start with a sprint [4].

As the importance of sprint ability emerges in the starting section, research on the effect of improving sprint ability is ongoing [5]. Among them, methods for improving sprint ability are presented in a short period of time in the field, not in continuous training methods. Static and dynamic stretching not only positive studies on improving sprint ability [6], but also negative results that not only do not improve sprint ability, but also repetitive sprint ability and jump ability [7]. As a result of research showing that it is more effective in flexibility [8] and jump ability [7] than sprint, opinions on sprint ability are divided. There are also many studies on the cooling effect on the sprint ability, compared to the control group that did nothing, cooling was effective in improving the sprint capacity [9], but the negative result was that cooling has a high temperature environmental effect and has no effect on the sprint ability [10].

Additional, a whole body vibration (WBV) response method has been proposed as another method for improving sprint ability. WBV is a mechanical stimulus characterized by vibrational motion that determines the amplitude (mm) while determining the repetition rate frequency (Hz) of the vibration [4]. When applying WBV to healthy adult males, the jump height was significantly higher [11], and also several studies reported that not only the 10m and 20m sprint speeds, but also the 30m sprint speed and 40m speed were faster [12–14]. Blood lactate levels, body and leg muscle fatigue and heart rate were also decreased more rapidly [15].

Research and application of sprint ability for bobsled-skeleton athletes are insufficient. In addition, there is only one study on Korean national athletes participating in the 2018 PyeongChang Olympics for the purpose of studying time reduction and improving performance. Therefore, this study aims to find out whether WVB has an effect on the sprint ability to shorten the time of bobsled-skeleton athletes.

## 2. Materials and methods

### 2.1. Study participation

Seventeen bobsled-skeleton athletes (male = 11, female = 6) were recruited from Korea Winter Olympics National Team. All participants who participated in the 2018 PyeongChang Winter Olympics. This study was approved by the Institutional Review Board at Korea Institute of Sport Science, Seoul, South Korea.

All participants who agreed to participate in the study had the study explained to them to ensure a complete understanding of its purpose and methods, in accordance with the ethical principles of the Declaration of Helsinki. The participants also signed an informed consent form before participation.

Physical characteristics of participants are listed in Table 1.

**Table 1:** The characteristic of the subjects.

Variables	Male (n = 11)	Female (n = 6)	Total (n = 17)
Age (years)	24.91 ± 3.24	21.17 ± 2.48	23.59 ± 3.45
Height (cm)	182.4 ± 7.48	167.9 ± 3.67	177.3 ± 9.50
Weight (kg)	97.75 ± 9.90	71.20 ± 5.08	88.38 ± 15.5
BMI (kg/m^2^)	29.71 ± 1.78	25.27 ± 1.20	28.14 ± 2.68

### 2.2. Study design

This study was a randomized crossover design in which participants completed two protocols. The participants were randomly assigned to either a sprint immediately after whole body vibration (WBV) or a sprint without whole body vibration (Non-WBV) protocol, during two separate visits by a period of 3 months. First visited, each participant’s given their informed consents and WBV or Non-WBV protocols performed. After 3 months, second visited participants in the WBV or Non-WBV protocols performed.

### 2.3. Measurement of body composition

The body composition variables were measured. Body mass and height were measured to the nearest 0.1 kg and 0.1 cm, respectively, using a body composition analyzer (Inbody 720, Body Composition Analyzer; Biospace, Seoul, Korea). Body mass index (BMI) was calculated as weight in kilograms divided by height in meters squared.

### 2.4. Sprint test

Four times 60-m sprint were performed. It was conducted two times Non-WBV and two times WBV to recorded the highest. Splits were measured at 15, 30, 45, 50, and 60 m and recorded to the nearest 0.01 second using a laser device focused on the bottom of the athletes back by an experienced operator (Witty, Microgate, Bolzano, Italy).

### 2.5. Whole Body Vibration (WBV)

Athletes were exposed to vertical sinusoidal WBV of 30 Hz with a 30 seconds (Galileo®, Novotec Medical, Germany). For the WBV, athletes stood with heel elevated and only their toes on the vibration platform, the knee at 100° flexion and leaning slightly forward.

### 2.6. Statistical analysis

All results are reported as the mean ± standard deviation. All data were analyzed using SPSS version 25.0 (SPSS Inc., Chicago, IL, USA). A comparison of sprint recorded between WBV and Non-WBV was analyzed by paired t-test. Statistical significance was accepted at a = 0.05.

## 3. Results

### 3.1. Compare sprint section records

Table 2 shows the sprint section records of males and females. In males, at the 45m (p<0.05) significant faster after WBV more than Non-WBV. In females, at the 15m (p<0.05), 30m (p<0.01), 45m (p<0.05), and 60m (p<0.05) significant faster after WBV more than Non-WBV.

**Table 2:** The participants’ sprint section record.

Variables		Sprint section record				
		15m	30m	45m	50m	60m
Male (n = 11)	WBV	2.25 ± 0.06	1.63 ± 0.05	1.57 ± 0.04	0.51 ± 0.03	1.08 ± 0.04
	Non-WBV	2.28 ± 0.08	1.67 ± 0.05	1.62 ± 0.08	0.54 ± 0.03	1.11 ± 0.07
	p-value	0.100	0.052	0.029	0.071	0.227
Female (n = 6)	WBV	2.58 ± 0.09	1.93 ± 0.06	1.91 ± 0.07	0.64 ± 0.02	1.31 ± 0.05
	Non-WBV	2.63 ± 0.10	2.00 ± 0.07	1.97 ± 0.09	0.66 ± 0.03	1.37 ± 0.06
	p-value	0.042	0.004	0.021	0.058	0.010

### 3.2. Compare sprint cumulative records

**Fig 1:**
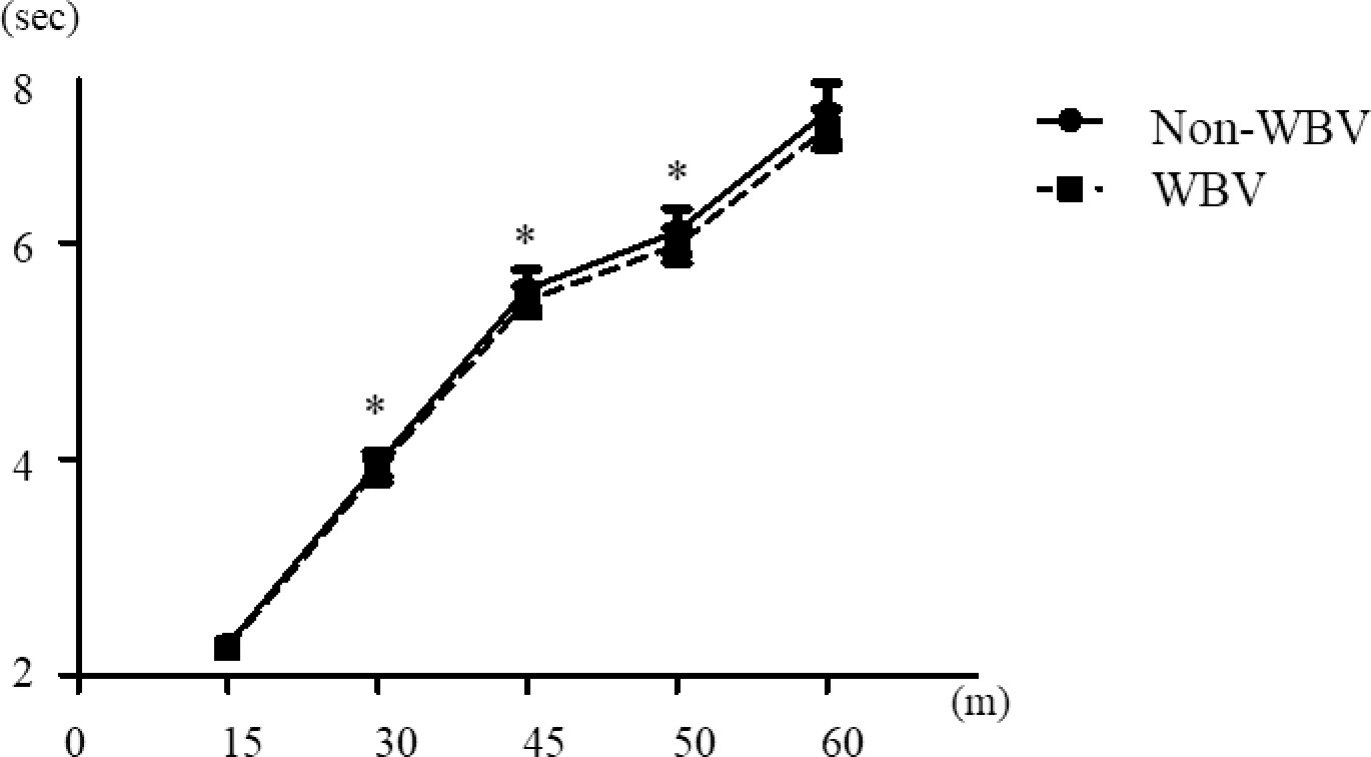
Sprint cumulative record of males. Data in the graph represent the means ± standard deviation. * Analyzed by paired t-test, * p<0.05.

**Fig 2:**
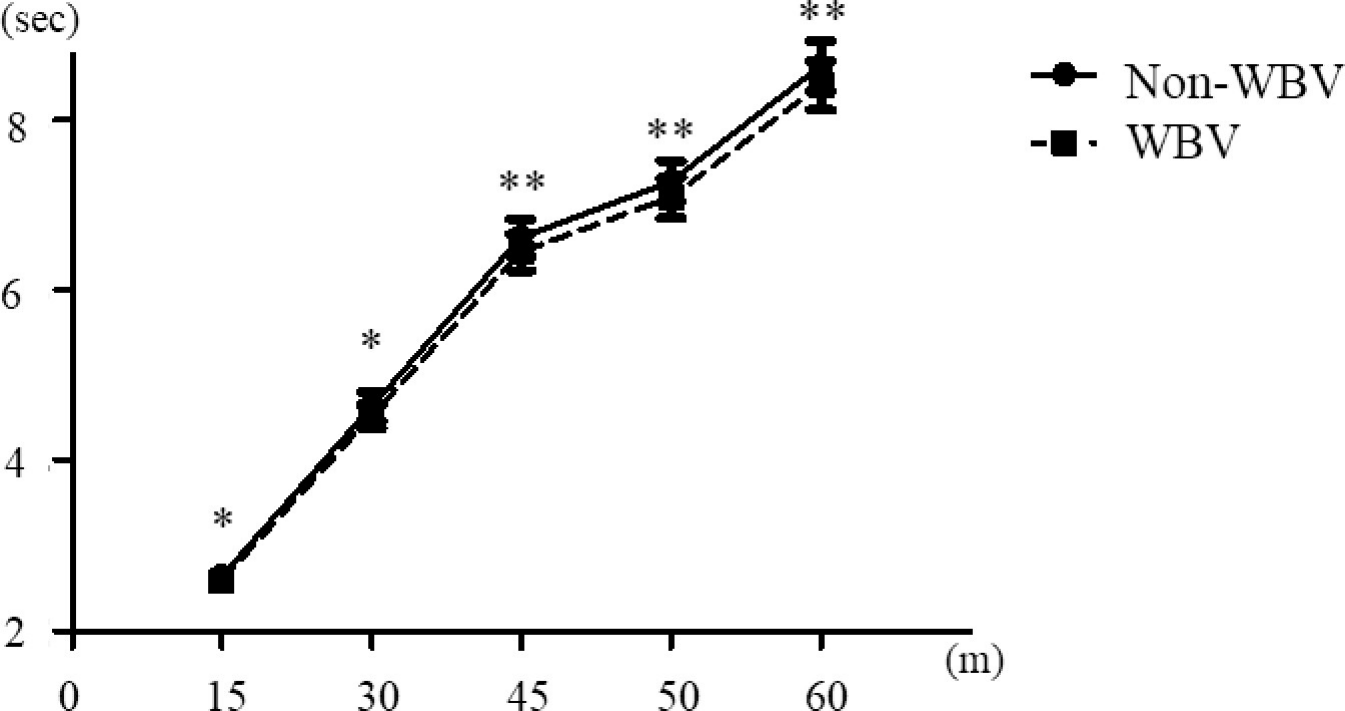
Sprint cumulative record of females. Data in the graph represent the means ± standard deviation. * Analyzed by paired t-test, * p<0.05, ** p<0.01.

Figs 1 and 2 shows the sprint cumulative records of males and females. In males, at the 30m (p<0.05), 45m (p<0.05), and 50m (p<0.05) significant faster after WBV more than Non-WBV (Fig 1). In females, at the 15m (p<0.05), 30m (p<0.05), 45m (p<0.01), 50m (p<0.01), and 60m (p<0.01) significant faster after WBV more than Non-WBV (Fig 2).

## 4. Discussion

In the current study, we investigated WVB has an effect on the sprint ability to shorten the time of bobsled-skeleton athletes. The main finding of the study that significant faster after WBV more than Non-WBV in males and females bobsled-skeleton athletes.

One of the many factors influencing sledding events such as bobsleigh-skeleton success and finish time is push start section [16]. According to the standard of the bobsled-skeleton track, the inclined part of the track where the athlete performs a push start is the start ramp section, consisting of a 2 degree downhill 15m section and a 50m of track designer-selected downhill, and passing sequentially in each section of 15m and 65m for time measurement [17]. The push start time is the time taken to move 50m of the measuring section of 15m and 65m [17]. Therefore, the push start time of the start ramp section is to sprint as fast as possible up to 65m. In this study, the sprint speed of the athletes in the 15m, 30m, 45m, 50m, and 60m sections was measured in more detail. Furthermore, we investigate the change after conducting WBV for sprint improvement in important start section.

To the best of our knowledge, this study included Korean bobsled-skeleton athletes who have participated in the only Winter Olympics among similar studies performed to date and therefore provides useful insight into the role of the WBV in trained individuals. In female athletes who performed WBV showed significantly faster sprinter ability in each section (15m, 30m, 45m, 50m and 60m) than female athletes who did not perform WBV. In addition, for male athletes who performed WBV showed significantly faster sprinter ability in 30m, 45m and 50m. During WBV, the individual’s body is exposed to low or high frequency-amplitude mechanical stimuli through a vibrating platform [18]. Vibration stimulates muscle spindles and transmits nerve impulses to initiate muscle contraction according to the tonic vibrational reflex (TVR) [19]. Muscles are fully activated through contraction may lead to motor unit activated, and consequently an increase in the strength of the muscles [20]. Impact of WBV on muscle activity, which recommended that a standing on the WBV platform for a long time results in full motor unit activation [18]. Tapp & Signorile reported that WBV may not be an effective to aerobic capacity, but could have a positive impact on lower body strength [21]. Pistone et al. reported that 4-weeks of WBV effective improving muscle strength of the knee flexor muscles [22].

Neuromuscular activity during vibration was improved compared to synchronous vibration, and neuromuscular activity during WBV was found to be closely related to the frequency of vibrations [23]. Cardinale & Lim reported that 30 Hz frequency is the highest reflex response in the muscles during WBV more than no vibrations, 40 Hz, and 50 Hz [24]. Ritzmann et al reported 30 Hz is effective for a WBV-based training regimen to achieve high activation intensity in the lower extremity muscles more than 5 Hz, 10 Hz, 15 Hz, 20 Hz, and 25 Hz [23]. Because vibrational energy is dissipated by the calf muscles as well as the ankle and knee joints, the proximity of the muscles to the vibrational stimulus might be affect the magnitude of the neuromuscular response to WBV exposure [25]. In this study, the knee at 100° flexion and leaning slightly forward position on the vibration platform was set at 30 Hz with a 30 seconds. It might be WBV helped athletes improve lower extremity muscle function with similar results to previous studies.

Finally, this study meaningfully conducted on the Korea national team athletes who participated in the 2018 PyeongChang Olympics. However, the present study had some limitations. The sample size was small, which limits our ability to determine the significance of the results. Therefore, additional studies with larger sample sizes and control group are required to determine the effectiveness of WBV. Another limitation was that various frequency in the WBV were not analyzed. Therefore, it will be necessary to observe changes in future studies by setting different frequencies and times.

## 5. Conclusions

In conclusion, in female athletes who performed WBV showed significantly faster sprinter ability in each section (15m, 30m, 45m, 50m and 60m) than female athletes who did not perform WBV. In addition, for male athletes who performed WBV showed significantly faster sprinter ability in 30m, 45m and 50m.

Thus, the results of this study indicated that significant faster after WBV more than Non-WBV in males and females bobsled-skeleton athletes.
